# Mediation of macronutrients and carbon by post-disturbance shelf sea sediment communities

**DOI:** 10.1007/s10533-017-0350-9

**Published:** 2017-06-12

**Authors:** Rachel Hale, Jasmin A. Godbold, Marija Sciberras, Jessica Dwight, Christina Wood, Jan G. Hiddink, Martin Solan

**Affiliations:** 1 School of Ocean and Earth Science, National Oceanography Centre Southampton, University of Southampton Waterfront Campus, European Way, Southampton, SO14 3ZH UK; 2 Biological Sciences,Faculty of Natural and Environmental Sciences, University of Southampton, Highfield Campus, Life Sciences Building 85, Southampton, SO17 1BJ UK; 3 School of Ocean Sciences, Bangor University, Menai Bridge, Bangor, LL59 5AB UK

**Keywords:** Bioturbation, Biodiversity, Ecosystem function, Nitrogen, Biogeochemical cycles, Recovery

## Abstract

Benthic communities play a major role in organic matter remineralisation and the mediation of many aspects of shelf sea biogeochemistry. Few studies have considered how changes in community structure associated with different levels of physical disturbance affect sediment macronutrients and carbon following the cessation of disturbance. Here, we investigate how faunal activity (sediment particle reworking and bioirrigation) in communities that have survived contrasting levels of bottom fishing affect sediment organic carbon content and macronutrient concentrations ([NH_4_–N], [NO_2_–N], [NO_3_–N], [PO_4_–P], [SiO_4_–Si]). We find that organic carbon content and [NO_3_–N] decline in cohesive sediment communities that have experienced an increased frequency of fishing, whilst [NH_4_–N], [NO_2_–N], [PO_4_–P] and [SiO_4_–Si] are not affected. [NH_4_–N] increases in non-cohesive sediments that have experienced a higher frequency of fishing. Further analyses reveal that the way communities are restructured by physical disturbance differs between sediment type and with fishing frequency, but that changes in community structure do little to affect bioturbation and associated levels of organic carbon and nutrient concentrations. Our results suggest that in the presence of physical disturbance, irrespective of sediment type, the mediation of macronutrient and carbon cycling increasingly reflects the decoupling of organism-sediment relations. Indeed, it is the traits of the species that reside at the sediment–water interface, or that occupy deeper parts of the sediment profile, that are disproportionately expressed post-disturbance, that are most important for sustaining biogeochemical functioning.

**Electronic supplementary material:**

The online version of this article (doi:10.1007/s10533-017-0350-9) contains supplementary material, which is available to authorized users.

## Introduction

Shelf seas are an important global resource that provide many benefits and ecosystem services to people, including nutrient cycling, carbon storage and food security (Worm et al. 2006), but human activity has led to degradation of many benthic habitats (Halpern et al. 2008). In particular, bottom fishing—the use of towed nets and dredges—causes surface and sub-surface physical disturbance that results in a range of morphological and biogeochemical changes in continental shelf and slope systems (Kaiser et al. 2000; Puig et al. 2012; Sciberras et al. 2016) depending on fishing type and frequency (Oberle et al. 2016), including sub-lethal injury or mortality of benthic invertebrates and the destruction of specific biogenic habitats (Kaiser et al. 2006; Cook et al. 2013). Bottom fishing practices may remove surficial sediments and mix organic matter into subsurface sediment layers where they can become buried (Duplisea et al. 2001; Warnken et al. 2003; O’Neill and Summerbell 2011). If burial of surficial sediments is below the oxic zone, organic matter is lost before aerobic remineralisation can take place (Mayer et al. 1991; Pilskaln et al. 1998) leading to increased anaerobic remineralisation through sulphate reduction (Duplisea et al. 2001; Trimmer et al. 2005). In addition, resuspension of sediments when fishing gears contact the sea bed increase both overlying water turbidity (Bradshaw et al. 2000; O’Neill and Summerbell 2011; O’Neill and Summerbell 2016; Martin et al. 2014) and suspended particulate organic matter concentration, which can modify phytoplankton production in shallow shelf seas (Riemann and Hoffmann 1991; Pilskaln et al. 1998; Palanques et al. 2001) and, subsequently, the remineralization of particulate organic matter to dissolved inorganic carbon, nitrogen and phosphorous. Associated changes in grain size distribution, increased sediment sorting and alterations to porosity (Trimmer et al. 2005) can disrupt nitrification and denitrification processes (Rysgaard et al. 1994) through changes in oxygen penetration (Warnken et al. 2003), which, in turn, may also affect faunal and microbial activity (Sciberras et al. in review). Over extended periods of time these physical and biogeochemical changes reduce habitat complexity (Kaiser et al. 2002) and alter community structure by reconfiguring species and functional trait dominance (Kaiser et al. 2006; Pusceddu et al. 2014; Sciberras et al. 2016), causing a shift from sessile emergent species with high biomass to smaller bodied infaunal species (Kaiser et al. 2000). Importantly, such selective forcing may skew trophic structure (Duffy 2003; Wohlgemuth et al. 2016) and lead to the loss of species interactions that influence nutrient generation and dynamics (Gilbertson et al. 2012); the active redistribution of particles and fluids by infaunal invertebrates, for example, directly contributes to the spatial and temporal heterogeneity of oxic and anoxic zones (Bertics and Ziebis 2009), the availability of organic matter (Levin et al. 1997), and the distribution of metabolic electron acceptors (Aller 1982; Fanjul et al. 2007) that are important in controlling microbial process rates and benthic-pelagic coupling linked to primary productivity (Lohrer et al. 2004).

Whilst the susceptibility of benthic communities and habitat integrity to perturbation associated with bottom fishing activity has long been established (Thrush and Dayton 2002; Jones 1992), evaluations of the longer term effects of restructured post-disturbance communities inhabiting altered sedimentary conditions on biogeochemical processes have been less prominent (Lambert et al. 2014). Cohesive shelf sediments have been considered to be particularly important areas for biogeochemical activity owing to the presence of elevated concentrations of organic matter and living biomass, contributing an estimated 44% of global denitrification and >40% of total organic matter burial (Muller-Karger et al. 2005; Seitzinger et al. 2006; Middleburg and Levin 2009). However, non-cohesive sediments also play a major role in the turnover of particulate organic matter, despite comparatively lower levels of living biomass, because of the influence of advective processes (Rao et al. 2007, 2008). These mechanistic differences mean that in cohesive sediments the remineralisation of organic carbon and generation of macronutrients is largely driven by the reduction of solutes, such as nitrate and sulphate, whilst in non-cohesive sediments remineralisation processes reflect the extent of advective porewater flows (Rocha 2008). However, sediment or habitat type can be a poor predictor of biogeochemical performance (Dernie et al. 2003) because the active redistribution of particles and fluids by infaunal macro-invertebrates disproportionately influences benthic fluxes and total benthic metabolism (Banta et al. 1999; Mermillod-Blondin et al. 2004). Hence, the level of biogeochemical performance that is realised will depend, at least in part, on the structure and composition of the post-disturbance surviving community (Solan et al. 2004a; Thomsen et al. 2017; Wohlgemuth et al. 2017). Here, we investigate whether post-disturbance changes in epifaunal and infaunal assemblage structure result in associated changes in organic carbon and nutrient cycling along gradients of chronic fishing activity in fishing grounds that contrast in sediment type. Our a priori assumption was that benthic macrofaunal communities would be restructured by chronic physical disturbance and that the adjusted post-disturbance community would persist long after the perturbation event (van Colen et al. 2012). Consequently, the relative role of these surviving communities in moderating organic carbon and macronutrient dynamics will be context specific and relate to the introduction, removal or rebalancing of traits that directly influence the processes that govern remineralisation, advection, resuspension and burial within environmental context (Godbold and Solan 2009).

## Materials and methods

**Fig. 1:**
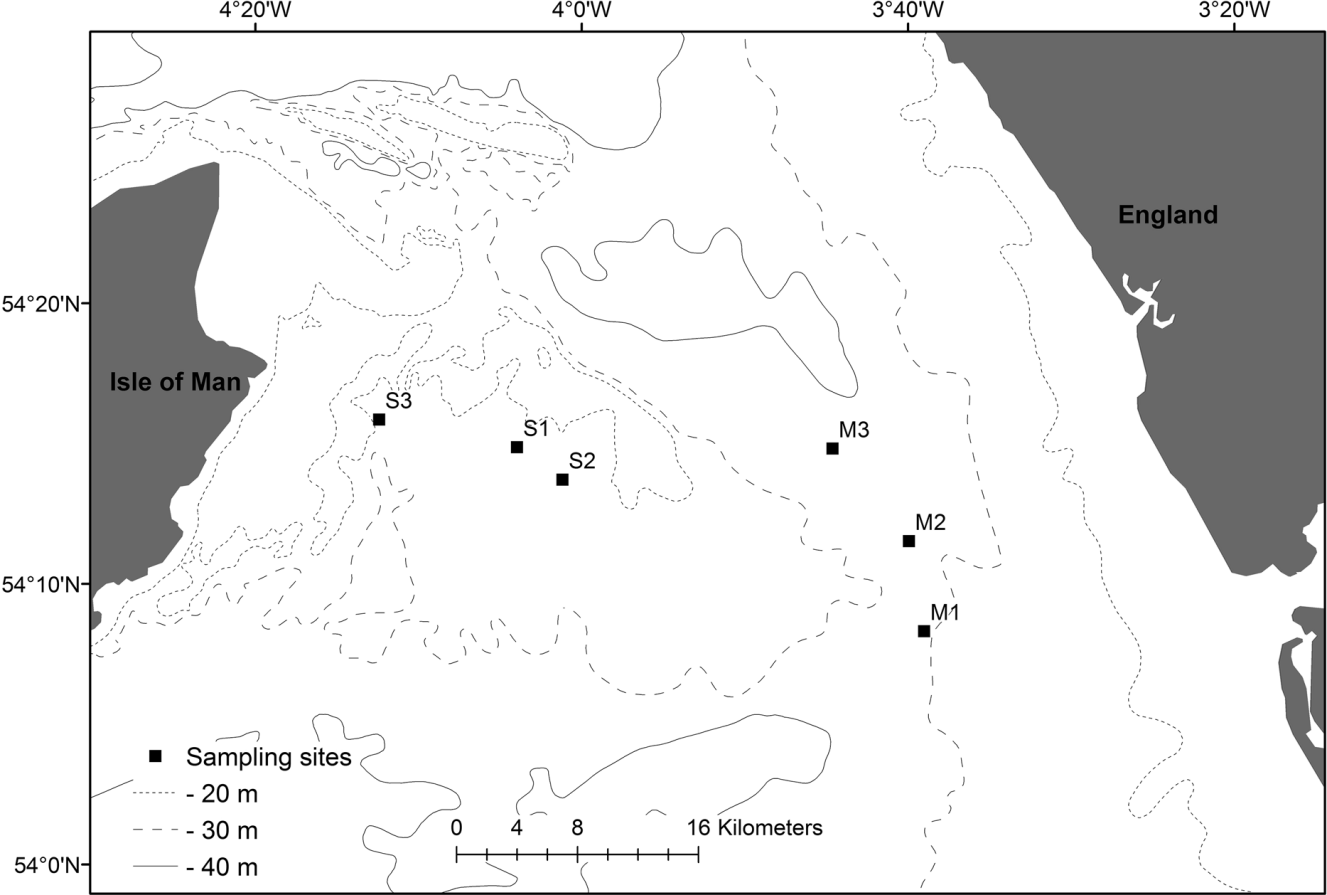
The location of study sites in the Irish Sea. Site characteristics are listed in Table 1 and station co-ordinates are listed in Supplementary Tables S1–S2. Sediment type is denoted as: *M* cohesive (muddy) sediments; or *S* non-cohesive (sandy) sediments. The frequency of fishing is: *1* low; *2* medium; or *3* high

To quantify the effects of chronic physical disturbance on macrofaunal community bioturbation (particle reworking, ventilation and bioirrigation), sediment organic carbon content and macronutrients, we focussed our study within the context of two important commercial fisheries located in the Irish Sea: (1) Norwegian Lobster (*Nephrops norvegicus)* grounds located in areas of cohesive (muddy) sediment that are fished using otter trawl nets with bobbin, roller and chain ground gear (sub-surface level impacts ≤35 cm; Eigaard et al. 2015) and, (2) Scallop (*Pecten maximus* and *Aequipecten opercularis*) fishing grounds located in areas with non-cohesive (sandy) sediment that are fished using otter trawl nets and dredges with chains and a toothed beam (sub-surface level impacts ≤15 cm; Eigaard et al. 2015). Following Sciberras et al. (2016), we categorised fishing activity in each area by computing the accumulated swept areas within a year from UK registered vessels (>15 m long) that use bottom-contact fishing gear over a 3 year period (sandy area: January 2009–December 2011, muddy area: January 2010–December 2012) to calculate the total seabed area swept (km^2^) by a fishing gear per annum. Specifically, we focussed on six locations that contrast in fishing regime (Table 1; Fig. 1): three in cohesive sediment (Supplementary Table S1, mean grain size < 63 μm, Supplementary Figure S1, location: off the coast of Cumbria, England, disturbance intensities—low: 3.8 times annum^−1^, medium: 5.9 times annum^−1^, high: 8.4 times annum^−1^, where disturbance refers to the frequency of bottom trawling or dredging of an area per year, otter trawled for *Nephrops norvegicus* and gadoid fish year round; Hinz et al. 2009); and three in non-cohesive sediment (Supplementary Table S2, mean grain size 63–500 μm, Supplementary Figure S2, location: off the east coast of the Isle of Man, fishing intensities—low: 0.25 times annum^−1^, medium: 0.51 times annum^−1^, high: 1.63 times annum^−1^, dredged for *Pecten maximus* May to November and otter trawled for *Aequipecten opercularis* June to October; Murray et al. 2010; Dignan et al. 2014). Variation in habitat characteristics were minimised to ensure that any observed differences reflected disturbance associated changes in species composition rather than environmental variability (Table 1).

**Table 1:** Representative site information after Sciberras et al. (2016)

Station	Sediment	Depth (m)	Tide stress (N m^−2^)	Wave stress (N m^−2^)	Fishing frequency (annum^−1^)	Fishery type
M1	Mud	26.0	0.17	0.69	3.8	Lobster
M2	Mud	28.0	0.16	0.52	5.9	Lobster
M3	Mud	28.5	0.22	0.68	8.4	Lobster
S1	Sand	19.8	0.17	1.00	0.25	Scallop
S2	Sand	18.3	0.17	1.14	0.51	Scallop
S3	Sand	18.8	0.11	0.73	1.63	Scallop

See Supplementary Figures S1 and S2 for sediment particle size detail and Supplementary Tables 1 and 2 for station location co-ordinates

At each site, five replicate intact sediment subsamples (LWH: 20 × 20 × 12 cm) were taken from 0.08 m^2^ NIOZ (Netherlands Institute for Sea Research, Texel) sediment cores collected from the RV Prince Madog (cruise: 22–28 June 2016), transferred to square transparent acrylic aquaria (LWH: 20 × 20 × 34 cm) and overlaid by ~20 cm (8L) of ambient seawater (salinity 33). Sediment surface scrapes were taken to determine sediment particle size (Malvern Mastersizer 2000, Malvern Instruments; Supplementary Figures S1 and S2) and percentage organic carbon content (loss on ignition, 375 °C, 1 h, Pansu and Gautheyrou 2006; org-C). Overlying seawater was replaced after 24 h to remove excess nutrients associated with assembly (Hale et al. 2014). Aquaria were maintained at ambient bottom temperature (Irish Sea, June 2016), 14 °C, in the dark and continually aerated at the School of Ocean Sciences, Bangor University for 7 days.

Faunal mediated sediment particle reworking was estimated non-invasively using fluorescent sediment profile imaging (f-SPI, following Solan et al. 2004b; Canon 400D, 10 s exposure, aperture f5.6, ISO 400, 3888 × 2592 pixels, effective resolution 39 × 39 μm pixel^−1^). Redistribution of fluorescent tracers (215 g aquarium^−1^, green colour, size class: <125 μm, mean particle size ~80 μm; Brian Clegg Ltd., UK) was determined from stitched composite images (four sides aquarium^−1^; RGB colour, JPEG compression) obtained under UV light illumination (Schiffers et al. 2011) after 6 days. The maximum vertical deviation of the sediment–water interface (upper—lower limit = surface boundary roughness, SBR) provided an indication of surficial activity. Following Hale et al. (2014), the mean (^f-SPI^L_mean_, time dependent indication of mixing), median (^f-SPI^L_med_, short-term depth of mixing), and maximum (^f-SPI^L_max_, full extent of vertical mixing over the long-term) depths of particle redistribution were calculated using a custom-made semi-automated macro that runs within FIJI (Schindelin et al. 2012; ImageJ, Version 1.47v).

Absolute concentrations of ammonium ([NH_4_–N]), nitrite ([NO_2_–N]), nitrate ([NO_3_–N]), phosphate ([PO_4_–P]) and silicate ([SiO_4_-Si]) were quantified (Technicon segmented flow colorimetric auto-analyser) in seawater samples (30 mL, 0.45 μm filtered) taken on day 5. Bioirrigation was quantified (Tecator flow injection auto-analyser, FIA Star 5010 series) from absolute changes (over 6 h) in the concentration of the inert tracer sodium bromide (Δ[Br^−^], mg L^−1^; applied concentration: 8.231 g sodium bromide, equivalent to 10 mM concentration; negative values indicate increased bioirrigation activity; Forster et al. 1999).

The sediment and associated macrofauna community (retained on 500 μm sieve) in each aquarium were fixed in a 10% formalin (4% formaldehyde) solution buffered with seawater (salinity, 33) on Day 7. Species were identified to the lowest possible taxon (94% to species, excluding Nematoda, which made up 36% of faunal abundance) and enumerated. For the estimation of biomass, fauna were blotted dry on an absorbent paper to remove excess liquid prior to wet weighing. All bivalves and gastropods were weighed within their shell. All tube dwelling worms (e.g. *Lanice conchilega*, *Owenia fusiformis*) were removed from their tubes prior to weighing.

As the two fisheries under study use different gears and operate in distinct habitat types with taxonomically different communities (Supplementary Figure S3), it was not possible to distinguish the effects of sediment type from the effects of fishing frequency within a single statistical analysis. Instead, single factor linear models were generated to examine the effect of bottom fishing disturbance for the dependent variables (SBR, ^f-SPI^L_mean_, ^f-SPI^L_med_, ^f-SPI^L_max_, Δ[Br^−^], org-C, [NH_4_–N], [NO_2_–N], [NO_3_–N], [PO_4_–P], [SiO_4_–Si]) within each fishery. Where variance was heterogeneous, a generalised least squares fitting approach (Pinheiro and Bates 2000; West et al. 2007) was applied (library: nlme; Pinheiro et al. 2016). In the non-cohesive sediments, we removed one outlier (associated with presence of a single individual of the Brachyuran crab *Goneplax rhomboides*) from the analysis ([NH_4_–N]: Cooks distance 0.93, [NO_2_–N]: Cooks distance 0.98, and [NO_3_–N]: Cooks distance 0.75). These analyses were conducted in R (R Core Team 2016; Version 3.3.1) and supplementary model (SM) information (the initial linear regression model, the minimal adequate model with GLS estimation, and a summary of the coefficient table) is included in the Supplementary Material. Community differences associated with the disturbance regime within each sediment type were determined graphically using non-metric multidimensional scaling (MDS) and quantified using Analysis of Similarities (ANOSIM) randomisation test based on abundance and biomass. The MDS procedure is based upon an iterative algorithm that repeatedly refines the distance between points in n-dimensional space until they fall into agreement with the similarity pattern for the same data. The distance in n-dimensional space that sample points deviate from the derived model forms a measure of the goodness of fit (“stress”, where a perfect representation has zero stress) of the resulting MDS plot (Clarke and Ainsworth 1993). MDS ordination representation stress values are indicated. The value of stress increases with reducing dimensionality of the ordination and lower values minimize misrepresentation (acceptable when <0.30). The relative contribution of individual species to the community was identified by calculating similarity percentages SIMPER (PRIMER+, Version 7). All data are available from the British Oceanographic Data Centre (Hale et al. 2017).

## Results

### Cohesive sediments

**Fig. 2:**
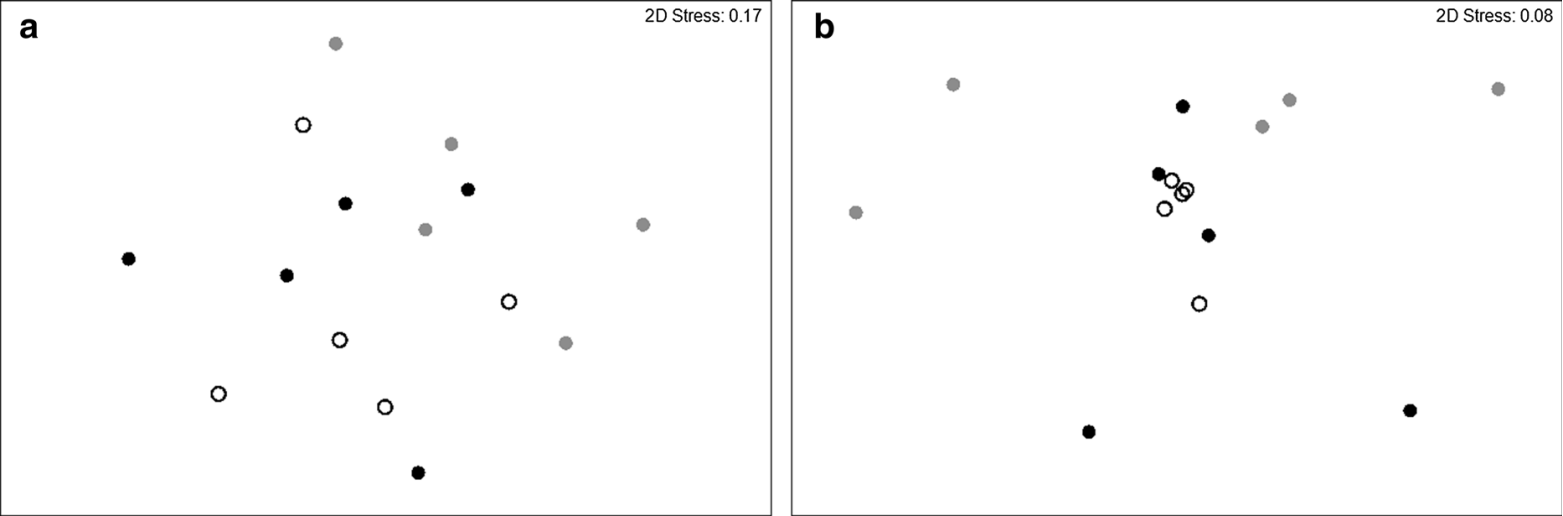
Non-metric two dimensional MDS configurations of log transformed Bray-Curtis similarity matrices of invertebrate **a** abundance, and **b** biomass for communities in cohesive sediment communities that have experienced contrasting levels of bottom fishing frequency (*open circle* low, *closed circle* medium, and *closed circle* high). MDS dimensionality representation stress values are **a** 0.17 and **b** 0.08

The number of species found at the cohesive sediment sites ranged from 5 to 17. Total species abundance and biomass ranged from 249 to 1020 individuals m^−2^ and 0.14 to 16.99 g m^−2^ respectively. Sites with different disturbance regimes did not differ in macrofaunal community abundance (nMDS, Fig. 2a; ANOSIM, p = 0.458), but did differ in macrofaunal community biomass (nMDS, Fig. 2b; ANOSIM, p = 0.013, Supplementary Table S3); the composition and total biomass of the species found at the site with a history of medium disturbance frequency was different from that found at the site with a history of low disturbance frequency (Mean biomass ± SD, n = 5: Site_med_, 4.08 ± 6.51 g m^−2^, Site_low_ 1.48 ± 0.51 g m^−2^; Statistic = 0.388, p = 0.008). Compositional differences between sites were largely associated with increased polychaete biomass (in particular, *Nephtys incisa* and *Notomastus laticerus*) at the site with a history of low disturbance frequency, the presence of a single individual of the decapod *Goneplax rhomboides* (1.29 g) at the site with a history of medium disturbance frequency (Supplementary Table S3).

The redistribution of sediment particles and fluids by the resident infauna were not affected by changes in the composition or structure of the community in cohesive sediment communities (SM S1-5). Surface boundary roughness (SBR) ranged from 0.84 to 6.25 cm, whilst the maximum vertical extent of mixing relative to the sediment–water interface (^f-SPI^L_max_) ranged from 1.05 to 8.26 cm (^f-SPI^L_mean_, 0.44 to 0.99 cm; ^f-SPI^L_med_, 0.24 to 0.71 cm) across all cohesive sediment locations.

**Fig. 3:**
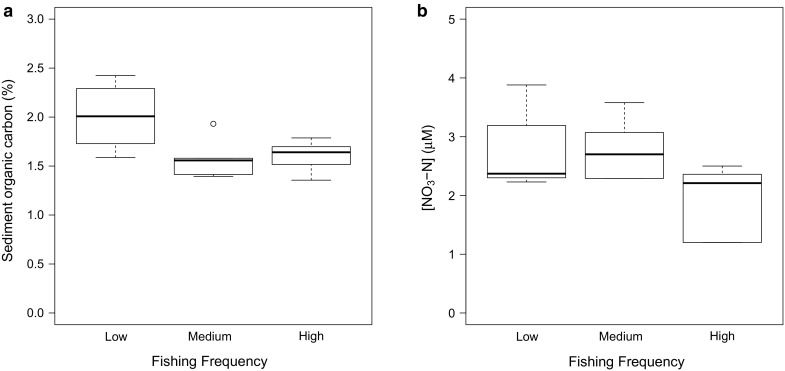
The effect of contrasting levels of bottom fishing frequency on **a** sediment organic carbon content (%) and **b** overlying [NO_3_–N] (μM) in cohesive sediment communities. In each case, the median is indicated at the midpoint, the upper and lower quartiles are indicated by the *hinges*, *lines* represent the *spread and open circles* indicate outliers

In communities with a history of a low frequency of fishing disturbance, mean sediment carbon content (org-C, ±SE; n = 5; 2.00 ± 0.16) was greater than in the sediments that experienced either a medium (coefficient ± SE = 0.43 ± 0.16, t = −2.64, p = 0.022) or high frequency of fishing disturbance (coefficient ± SE = 0.41 ± 0.16, t = −2.49, p = 0.028, Fig. 3a; SM S6). With the exception of [NO_3_–N] we found no evidence that overlying seawater nutrient concentrations ([NH_4_–N] range 5.11–20.91 μM, SM S7; [NO_2_–N] range 0.17–0.39 μM, SM S8; [PO_4_–P] range 0.17–0.91 μM, SM S10; and [SiO_4_–Si] range 13.77–30.90 μM, SM S11) were affected by any changes in the composition or structure of the community. Sediments with a history of lower and medium disturbance frequency had higher mean overlying [NO_3_–N] (±SE; n = 5; 3.05 ± 0.33 and 3.05 ± 0.24 μM, respectively) than those with a history of higher frequency disturbance (coefficient ± SE: −0.92 ± 0.41, t = 2.23, p = 0.045 and −0.9 ± 0.41, t = 2.22, p = 0.046, respectively; Fig. 3b, SM S9).

### Non-cohesive sediments

**Fig. 4:**
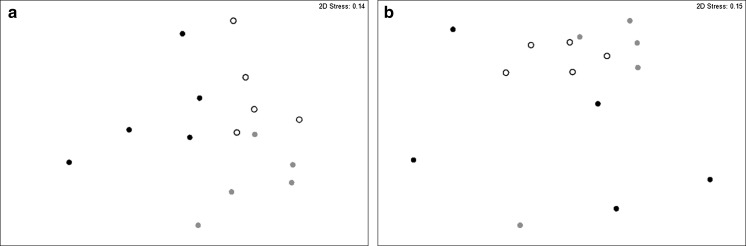
Non-metric two dimensional MDS configurations of log transformed Bray-Curtis similarity matrices of invertebrate **a** abundance, and **b** biomass for communities in non-cohesive sediment communities that have experienced contrasting levels of bottom fishing frequency (*open circle* low, *closed circle* medium, and *closed circle* high). MDS dimensionality representation stress values are **a** 0.17 and **b** 0.08

The number of species at sites with non-cohesive sediments ranged from 4 to 15. Species total abundance and biomass ranged from 87 to 1144 individuals m^−2^ and 0.12 to 406.79 g m^−2^ respectively. In non-cohesive sediments, community composition and abundance separated from one another with respect to disturbance frequency (nMDS, Fig. 4a; ANOSIM, p = 0.001; low:medium Statistic = 0.392, p = 0.008, low:high Statistic = 0.36, p = 0.032, medium:high Statistic = 0.52, p = 0.008). This delineation was largely due to a comparatively higher representation of polychaetes (*Poecilochaetus serpens*, *Magelona minuta*, and *Sthenelais limicola*) in communities with a history of low frequency disturbance; the polychaete *Lagis koreni* in communities with a history of low and medium frequency disturbance; Nematoda in communities with a history of medium frequency disturbance; and the polychaete *Ophelina acuminata* in communities with a history of high frequency disturbance (Supplementary Table S4). Similarly, the composition and total biomass of species within communities that experienced a history of low and medium frequency disturbance differed from those with a history of high frequency disturbance (nMDS, Fig. 4b; ANOSIM, p = 0.008; low:high Statistic = 0.312, p = 0.008, medium:high Statistic = 0.32, p = 0.024). This delineation relates to the presence of a razor clam, *Ensis ensis* (25.81 g) within the communities that have experienced medium frequency disturbance, a large urchin *Echinocardium cordatum* (13.46 g) and bivalve *Gari fervensis* (3.21 g) within the communities that have experienced high frequency disturbance, an increase in the biomass of the polychaete *Lagis koreni* from low to medium frequency disturbance, and the absence of several species in communities that have experienced low or medium frequency disturbance (Supplementary Table S5).

**Fig. 5:**
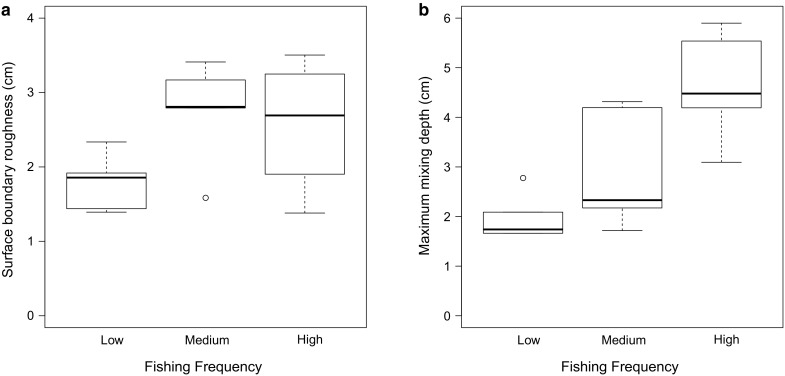
The effect of contrasting levels of bottom fishing frequency on **a** surface boundar roughness (cm) and **b** the maximum mixed depth of particle reworking (^f-SPI^L_max_, cm) in non-cohesive sediment communities. In each case, the median is indicated at the midpoint, the upper and lower quartiles are indicated by the *hinges*, *lines* represent the *spread and open circles* indicate outliers

The mean (^f-SPI^L_mean_, range 0.67–1.25 cm; SM S13) and median (^f-SPI^L_med_, range 0.64–1.10 cm; SM S14) depth of sediment reworking were unaffected by the composition or structure of the community. In contrast, mean (±SE; n = 5) surface boundary roughness (SBR; SM S12; Fig. 5a) was higher in communities that have experienced medium frequency disturbance (2.75 ± 0.31) relative to those that had experienced low frequency disturbance (coefficient ± SE = −0.96 ± 0.4, t = 2.19, p = 0.048). SBR was variable at the sites that had experienced a high frequency of disturbance, and did not differ from those observed at the low or medium frequency disturbance sites. Additionally, the mean (±SE; n = 5) maximum depth of sediment reworking (^f-SPI^L_max_; SM S15; Fig. 5b) in communities that have experienced high frequency disturbance (4.64 ± 0.50 cm) was deeper than in those that had experienced low (coefficient ± s.e. = −2.65 ± 0.63, t = 4.22, p = 0.001) or medium (coefficient ± SE = −1.69 ± 0.63, t = 2.70, p = 0.020) frequency disturbance. Bioirrigation (Δ[Br^−^]) did not change with disturbance history (SM S16).

**Fig. 6:**
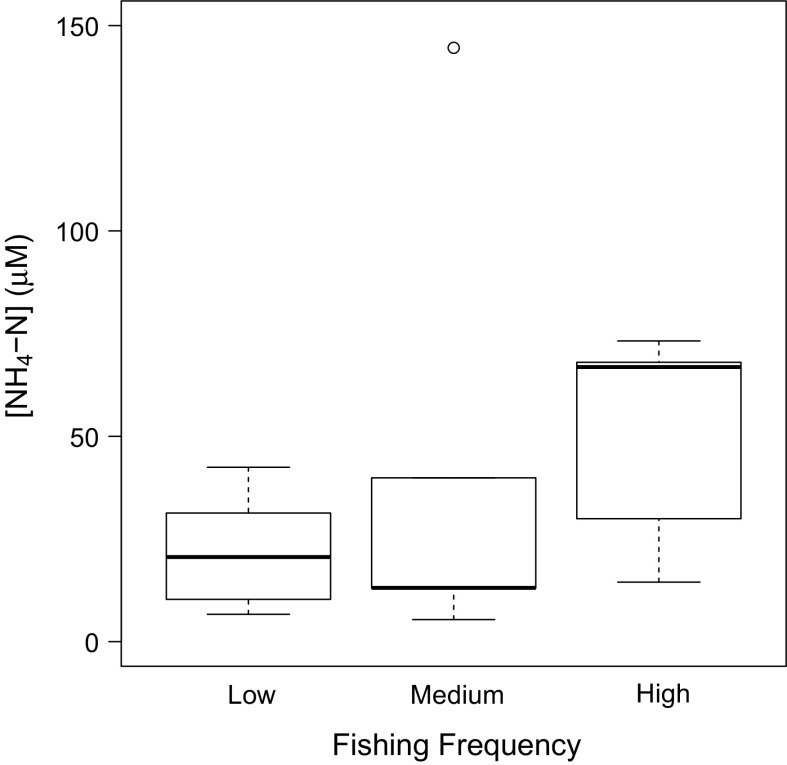
The effect of contrasting levels of bottom fishing frequency on overlying [NH_4_–N] (μM) in non-cohesive sediment communities. In each case, the median is indicated at the midpoint, the upper and lower quartiles are indicated by the *hinges*, *lines* represent the *spread and open circles* indicate outliers

Changes in the composition or structure of the community had no discernable effect on sediment carbon content (org-C; range, 0.23–0.51%; SM S17) in non-cohesive sediments. For macronutrients, with the exception of [NH_4_–N], we found no evidence that overlying seawater nutrient concentrations ([NO_2_–N], range 0.19–0.38 μM, outlier 1.76 μM, SM S19; [NO_3_–N], range 1.39–3.98 μM, outlier 13.28 μM, SM S20; [PO_4_–P], range 0.35–2.84 μM, SM S21; and [SiO_4_–Si], range 24.19–65.79 μM, SM S22) were affected by the disturbance history of the community. Sediments with a history of higher frequency disturbance had a larger mean (±SE; n = 5) overlying [NH_4_–N] (50.51 ± 26.51 μM) than those with a history of low and medium frequency of disturbance (Fig. 6, SM S18; (coefficient ± SE = −28.24 ± 12.62, t = 2.24, p = 0.047; coefficient ± SE = −32.66 ± 13.38, t = 2.44, p = 0.033 respectively).

## Discussion

Our study indicates a negative influence of chronic physical disturbance on the integrity of invertebrate communities, but reveals that the form and extent of restructuring is dependent on sediment type. In cohesive sediments, we find that there are few differences between sediment communities that have experienced different frequencies of fishing (as found in Pommer et al. 2016), but there are fundamental changes in species composition, abundance and biomass among post-fishing disturbance levels in non-cohesive sediments. Importantly, these alterations to infaunal biodiversity are not necessarily transformative in terms of biogeochemical functioning as theory might predict (Cardinale et al. 2012), rather there is little to differentiate the relative contribution of post-disturbance communities in moderating organic carbon and macronutrient dynamics (Wrede et al. 2017). Alterations in community biomass associated with fishing pressure, as observed here, are known to decrease the strength of interspecific interactions and minimise the effects of biodiversity on ecosystem processes (Caliman et al. 2012), yet the lowest species abundances documented here were not in communities that had experienced the greatest frequency of fishing. This apparently anomalous finding can be explained, however, because the areas under study likely represent a permanently disrupted state (Collie et al. 2000; Kaiser et al. 2000; Pommer et al. 2016), with recovery times measured in years (Kaiser et al. 2006). In such environments there tends to be a predominance of small species with opportunistic reproductive modes that are able to respond quickly post-disturbance and can reach high abundances, but these species are more likely to have a low bioturbation potential (Solan et al. 2004a; Queirós et al. 2013). As per capita effects on sediment–water nutrient fluxes are disproportionately greater for larger polychaetes (Bosch et al. 2015), there is less capacity for most of the resident infauna to influence biogeochemical processes. A similar effect is also true for non-cohesive sediments which, due to their inherent mobility, host a high proportion of opportunistic species (Collie et al. 2000), but these communities also harbour large deep-burrowing fauna (e.g. decapod crustaceans, spatangoid urchins) that can form extensive galleries (Lohrer et al. 2004) and disproportionately augment oxygen uptake (Volkenborn et al. 2012) and the flux of dissolved substances across the water–sediment interface (Osinga et al. 1995; Bird et al. 1999; D’Andrea and DeWitt 2009). The presence of these species, in particular the spatangoid *Echinocardium cordatum*, offers an explanation for the enhanced [NH_4_–N] observed in non-cohesive sediment communities that have experienced a high frequency of fishing activity.

 Based on our findings, a naïve hypothesis would be that the frequency of physical disturbance (here, bottom fishing) is less important for biogeochemical cycling in cohesive sediments than it is in non-cohesive sediments, but in the absence of a comparable fully functional ecosystem such a conclusion is premature (Thrush and Dayton 2002). Indeed, even a low frequency of disturbance can cause significant changes to the biotic and abiotic components of the system (Kaiser et al. 2000) and it is possible that intensive fishing disturbance can cause a reduction of [NO_3_–N] that relates to over-mobilisation of sediment, increased microbial activity, and net loss of N from the sediments (Bertics et al. 2010, Laverock et al. 2011, Mayer et al. 1998). Whilst it is clear that non-cohesive sediment communities are vulnerable to changes in the frequency of fishing, even though the fishing pressure is relatively low, near-bed current flows and the permeability of the sediment profile mean that organic carbon cycling is already rapid (Rocha 2008), making it difficult to assess how nutrient sediment–water exchange is affected as communities are modified. Cohesive sediments by contrast, are dominated by diffusive processes, where the remineralisation of organic matter is largely driven by the reduction of solutes (Kitidis et al. 2017). The decreasing pattern in [NO_3_–N] with increasing [NH_4_–N] observed here suggests that macrofaunal bioturbation did stimulate microbial denitrification. We also observed an increase in [NO_3_–N] within communities that had experienced the lowest frequency of fishing. As archaeal and bacterial denitrifiers and anammox transcripts are known to increase in communities that have been subject to greater disturbance by bottom fishing (Sciberras et al. in review), it is tempting to speculate that increased bioturbation activity in less impacted (more diverse) communities leads to a decline in [NO_3_–N] by increasing oxygen availability and stimulating, nitrification, however we did not observe any notable differences in bioturbation or community structure across the range of sediment communities.

Overall, our results emphasise the importance of benthic community composition and structure for sustaining biogeochemical condition in both cohesive and non-cohesive sediments. Importantly, the form and extent of community restructuring depends on the susceptibility of individual species within the community, differs between sediment type and with the characteristics of physical forcing (here, frequency and type of fishing). Our data indicate that species traits become skewed towards opportunistic lifestyles that have minimal capacity for influencing the post-disturbance recovery of biogeochemical condition and, under these circumstances organism-sediment relations that underpin the mediation of macronutrient and carbon cycling can become decoupled. However, our data indicate that the difference in the numbers of opportunistic species between low and high fishing frequency is greater in non-cohesive sediments. Whilst fishing pressure and other anthropogenic activities are increasingly relevant to the protection of natural capital and the sustainable management of ecosystem services (Ormerod and Carleton 2016), our findings suggest that management should focus on the connectivity between multiple factors that contribute to biogeochemical performance (Pittman and Armitage 2016). In particular understanding when and under what circumstances organism-sediment relations become decoupled will be of value in determining the mechanistic link between forcing, the selective alteration of biotic communities and the observed levels of ecosystem functioning.

## Electronic supplementary material

Below is the link to the electronic supplementary material.

Supplementary material 1 (DOCX 299 kb)

Supplementary material 2 (DOCX 99 kb)
