# Supplementary material for: Mediation of macronutrients and carbon by post-disturbance shelf sea sediment communities
Source: Biogeochemistry. 2017 Jun 12;135(1):121–33. doi: 10.1007/s10533-017-0350-9 (PMC6961522; doi:10.1007/s10533-017-0350-9)
Supplement: Supplementary file 1 — Supplementary material 1 (DOCX 299 kb) [file 10533_2017_350_MOESM1_ESM.docx]

**Supplementary Material Part 1 of 2**

**Title: Mediation of macronutrients and carbon by post-disturbance shelf sea sediment communities**

**Journal: Biogeochemistry**

**Authors: Rachel Hale, Jasmin A. Godbold, Jessica Dwight, Christina Wood, Marija Sciberras, Jan G. Hiddink, Martin Solan**

Affiliations:

Rachel Hale, Jasmin A. Godbold, Jessica Dwight, Christina Wood, Martin Solan

Ocean and Earth Science,

National Oceanography Centre Southampton,

University of Southampton Waterfront Campus,

European Way, Southampton, SO14 3ZH

Jasmin A. Godbold

Biological Sciences

Faculty of Natural & Environmental Sciences

Life Sciences Building 85

University of Southampton

Highfield Campus

Southampton, SO17 1BJ

Marija Sciberras, Jan Hiddink

School of Ocean Sciences,

Bangor University,

Menai Bridge, LL59 5AB

Correspondence address:

r.hale@soton.ac.uk

School of Ocean and Earth Science,

National Oceanography Centre Southampton,

University of Southampton Waterfront Campus,

European Way, Southampton, SO14 3ZH

**Supplementary Material**

Supplementary Table S1 | Cohesive site replicate sample location details collected on RV Prince Madog, cruise: 22-28 June 2016.

| Date | Time | Cast no | Latitude (N) | Longitude (E) | Fishing Frequency |
| --- | --- | --- | --- | --- | --- |
| 23.06.15 | 14:57 | 31 | 54.261 | -3.729 | High |
| 23.06.15 | 15:10 | 32 | 54.261 | -3.733 | High |
| 23.06.15 | 15:23 | 33 | 54.261 | -3.733 | High |
| 23.06.15 | 15:36 | 34 | 54.261 | -3.734 | High |
| 23.06.15 | 15:48 | 35 | 54.260 | -3.734 | High |
| 23.06.15 | 19:50 | 48 | 54.205 | -3.652 | Medium |
| 23.06.15 | 20:02 | 49 | 54.206 | -3.652 | Medium |
| 23.06.15 | 20:17 | 50 | 54.206 | -3.652 | Medium |
| 23.06.15 | 20:29 | 51 | 54.206 | -3.653 | Medium |
| 23.06.15 | 20:40 | 52 | 54.205 | -3.653 | Medium |
| 23.06.15 | 21:52 | 54 | 54.157 | -3.641 | Low |
| 23.06.15 | 22:03 | 55 | 54.156 | -3.635 | Low |
| 23.06.15 | 22:13 | 56 | 54.156 | -3.637 | Low |
| 23.06.15 | 22:25 | 57 | 54.156 | -3.633 | Low |
| 23.06.15 | 22:36 | 58 | 54.155 | -3.634 | Low |

Supplementary Table S2 | Non-cohesive site replicate sample location details collected on RV Prince Madog, cruise: 22-28 June 2016.

| Date | Time | Cast no | Latitude (N) | Longitude (E) | Fishing Frequency |
| --- | --- | --- | --- | --- | --- |
| 24.06.15 | 21:26 | 85 | 54.238 | -4.007 | Medium |
| 24.06.15 | 21:36 | 86 | 54.239 | -4.007 | Medium |
| 24.06.15 | 21:46 | 87 | 54.241 | -4.007 | Medium |
| 24.06.15 | 21:55 | 88 | 54.242 | -4.008 | Medium |
| 24.06.15 | 22:07 | 89 | 54.243 | -4.008 | Medium |
| 25.06.15 | 06:07 | 94 | 54.257 | -4.055 | Low |
| 25.06.15 | 06:27 | 95 | 54.252 | -4.056 | Low |
| 25.06.15 | 06:41 | 96 | 54.255 | -4.054 | Low |
| 25.06.15 | 06:50 | 97 | 54.257 | -4.053 | Low |
| 25.06.15 | 07:00 | 98 | 54.259 | -4.052 | Low |
| 25.06.15 | 08:10 | 100 | 54.269 | -4.188 | High |
| 25.06.15 | 08:18 | 101 | 54.268 | -4.195 | High |
| 25.06.15 | 08:27 | 102 | 54.269 | -4.194 | High |
| 25.06.15 | 08:38 | 103 | 54.271 | -4.193 | High |
| 25.06.15 | 08:48 | 104 | 54.272 | -4.193 | High |

Supplementary Figure 1 | Sediment particle size frequency distribution (n = 5) at the cohesive site with a) low, b) medium and c) high historic fishing frequencies showing the mean (± SD) particle size and particle size skewness (skew) and kurtosis (kurt) following Folk and Ward (Folk RL, Ward WC 1957 Brazos River bar: a study in the significance of grain size parameters. Journal of Sediment Petrol 27:3-26).

| a)   |
| --- |
| b)   |
| c)   |

Supplementary Figure 2 | Sediment particle size frequency distribution (n = 5) at the non-cohesive site with a) low, b) medium and c) high historic fishing frequencies showing the mean (± SD) particle size and particle size skewness (skew) and kurtosis (kurt) following Folk and Ward (Folk RL, Ward WC 1957 Brazos River bar: a study in the significance of grain size parameters. Journal of Sediment Petrol 27:3-26).

| a)   |
| --- |
| b)   |
| c)   |

Supplementary Figure 3 | Non-metric two dimensional MDS configurations of square root transformed Bray-Curtis similarity matrices of invertebrate (a) abundance, and (b) biomass for communities in cohesive (◆ and non-cohesive (⚫) sediment.

a)

b)

**Statistical model summaries**

Summary of the statistical analyses for our 22 statistical models in (A) cohesive (Models S1-S11) and (B) non-cohesive sediments (Models S12-S22). For each model, we list the initial linear regression model, the minimal adequate model with GLS estimation, and a summary of the coefficient table. The coefficients indicate the relative performance of each treatment level (Fishing Frequency: Low, Medium, and High) relative to the re-levelled baseline (as indicated, initial baseline: Low). Coefficients ± SE and t-values are presented alongside corresponding significance values (in parentheses). For intercept only models, data are presented as a boxplot for information only as we found no evidence of an effect of fishing frequency. In each case, the median is indicated at the midpoint, the upper and lower quartiles are indicated by the hinges, lines represent the spread and open circles indicate outliers.

**A. Cohesive sediments**

**Supplementary Model S1 | Surface boundary roughness (SBR, cm)**

Initial linear regression model:

lm(SBR ~ as.factor(Fishing Frequency))

No minimal adequate model, intercept only (Fishing Frequency, L-ratio = 3.53, d.f. = 4, p = 0.1713)

**Supplementary Model S2 | Mean mixed depth of particle redistribution (^f-SPI^L_mean_, cm)**

Initial linear regression model:

lm(^f-SPI^L_mean_ ~ as.factor(Fishing Frequency))

No minimal adequate model, intercept only (Fishing Frequency, L-ratio = 1.96, d.f. = 4, p = 0.3752)

**Supplementary Model S3 | Median mixed depth of particle redistribution (^f-SPI^L_med_, cm)**

Initial linear regression model:

lm(^f-SPI^L_med_ ~ as.factor(Fishing Frequency))

No minimal adequate model, intercept only (Fishing Frequency, L-ratio = 0.40, d.f. = 4, p = 0.8207)

**Supplementary Model S4 | Maximum mixed depth of particle redistribution (^f-SPI^L_max_, cm)**

Initial linear regression model:

lm(^f-SPI^L_max_ ~ as.factor(Fishing Frequency))

No minimal adequate model, intercept only (Fishing Frequency, L-ratio = 0.24, d.f. = 4, p = 0.8871)

**Supplementary Model S5 | Bioirrigation (Δ[Br^−^], mg L^-1^)**

Initial linear regression model:

lm(Δ[Br^−^] ~ as.factor(Fishing Frequency))

No minimal adequate model, intercept only (Fishing Frequency, L-ratio = 0.36, d.f. = 2, p = 0.8339)

**Supplementary Model S6 | Sediment percentage organic carbon content (org-C, %)**

Initial linear regression model:

lm(org-C ~ as.factor(Fishing Frequency))

Minimal adequate model:

gls(org-C ~ as.factor(Fishing Frequency),

method = "REML")

Coefficient table:

Intercept ± SE (when baseline is for Low fishing frequency): 2.01 ± 0.12, t = 17.33, p < 0.0001.

|  | Low | Medium | High |
| --- | --- | --- | --- |
| Low | - | -0.43 ± 0.16  -2.64  (0.022) | -0.41 ± 0.16  -2.49  (0.028) |
| Medium | 0.43 ± 0.16  2.64  (0.022) | - | 0.02 ± 0.16  0.15  (0.885) |
| High | 0.41 ± 0.16  2.49  (0.028) | -0.02 ± 0.16  -0.15  (0.885) | - |

**Supplementary Model S7 | Ammonium ([NH_4_-N], μM)**

Initial linear regression model:

lm([NH_4_] ~ as.factor(Fishing Frequency))

No minimal adequate model, intercept only (Fishing Frequency, L-ratio = 2.00, d.f. = 4, p = 0.3681)

**Supplementary Model S8 | Nitrite ([NO_2_-N], μM**

Initial linear regression model:

lm([NO_2_] ~ as.factor(Fishing Frequency))

No minimal adequate model, intercept only (Fishing Frequency, L-ratio = 0.69, d.f. = 4, p = 0.7079)

**Supplementary Model S9 | Nitrate ([NO_3_-N], μM)**

Initial linear regression model:

lm([NO_3_] ~ as.factor(Fishing Frequency))

Minimal adequate model:

lm([NO_3_] ~ as.factor(Fishing Frequency))

Coefficient table:

Intercept ± SE (when baseline is for Low fishing frequency): 2.79 ± 0.29, t = 9.74, p < 0.0001.

|  | Low | Medium | High |
| --- | --- | --- | --- |
| Low | - | -0.01 ± 0.41  -0.02  (0.985) | -0.90 ± 0.41  -2.22  (0.047) |
| Medium | 0.01 ± 0.41  0.02  (0.985) | - | -0.89 ± 0.41  -2.20  (0.048) |
| High | 0.90 ± 0.41  2.22  (0.047) | 0.89 ± 0.41  2.20  (0.048) | - |

**Supplementary Model S10 | Phosphate ([PO_4_-P], μM)**

Initial linear regression model:

lm([PO_4_] ~ as.factor(Fishing Frequency))

No minimal adequate model, intercept only (Fishing Frequency, L-ratio = 1.36, d.f. = 4, p = 0.5056)

**Supplementary Model S11 | Silicate ([SiO_4_-Si], μM)**

Initial linear regression model:

lm([SiO_4_] ~ as.factor(Fishing Frequency))

No minimal adequate model, intercept only (Fishing Frequency, L-ratio = 3.55, d.f. = 2, p = 0.1699)

**B. Non-cohesive sediments**

**Supplementary Model S12 | Surface boundary roughness (SBR, cm)**

Initial linear regression model:

lm(SBR ~ as.factor(Fishing Frequency))

Minimal adequate model:

gls(SBR ~ as.factor(Fishing Frequency),

method = "REML")

Coefficient table:

Intercept ± SE (when baseline is for Low fishing frequency): 1.79 ± 0.31, t = 5.76, p = 0.0001.

|  | Low | Medium | High |
| --- | --- | --- | --- |
| Low | - | 0.96 ± 0.44  2.19  (0.048) | 0.76 ± 0.44  1.72  (0.110) |
| Medium | -0.96 ± 0.44  -2.19  (0.048) | - | -0.21 ± 0.44  -0.47  (0.645) |
| High | -0.76 ± 0.44  -1.72  (0.110) | 0.21 ± 0.44  0.47  (0.645) | - |

**Supplementary Model S13 | Mean mixed depth of particle redistribution (^f-SPI^L_mean_, cm)**

Initial linear regression model:

lm(^f-SPI^L_mean_ ~ as.factor(Fishing Frequency))

No minimal adequate model, intercept only (Fishing Frequency, L-ratio = 3.06, d.f. = 2, p = 0.2164)

**Supplementary Model S14 | Median mixed depth of particle redistribution (^f-SPI^L_med_, cm)**

Initial linear regression model:

lm(^f-SPI^L_med_ ~ as.factor(Fishing Frequency))

No minimal adequate model, intercept only (Fishing Frequency, L-ratio = 4.57, d.f. = 4, p = 0.1017)

**Supplementary Model S15 | Maximum mixed depth of particle redistribution (^f-SPI^L_max_, cm)**

Initial linear regression model:

lm(^f-SPI^L_max_ ~ as.factor(Fishing Frequency))

Minimal adequate model:

gls(^f-SPI^L_max_ ~ as.factor(Fishing Frequency),

method = "REML")

Coefficient table:

Intercept ± SE (when baseline is for Low fishing frequency): 1.99 ± 0.44, t = 4.47, p = 0.0008.

|  | Low | Medium | High |
| --- | --- | --- | --- |
| Low | - | 0.96 ± 0.63  1.53  (0.152) | 2.65 ± 0.63  4.22  (0.001) |
| Medium | -0.96 ± 0.63  -1.53  (0.152) | - | 1.69 ± 0.63  2.70  (0.020) |
| High | -2.65 ± 0.63  -4.22  (0.001) | -1.69 ± 0.63  -2.70  (0.020) | - |

**Supplementary Model S16 | Bioirrigation (Δ[Br^−^], mg L^-1^)**

Initial linear regression model:

lm(Δ[Br^−^] ~ as.factor(Fishing Frequency))

No minimal adequate model, intercept only (Fishing Frequency, F = 1.71, d.f. = 12, p = 0.2217)

**Supplementary Model S17 | Sediment percentage organic carbon content (org-C, %)**

Initial linear regression model:

lm(org-C ~ as.factor(Fishing Frequency))

No minimal adequate model, intercept only (Fishing Frequency, L-ratio = 5.00, d.f. = 2, p = 0.0822)

**Supplementary Model S18 | Ammonium ([NH_4_-N], μM)**

Initial linear regression model:

lm([NH_4_] ~ as.factor(Fishing Frequency))

No minimal adequate model, intercept only (Fishing Frequency, L-ratio = 4.48, d.f. = 4, p = 0.1066)

Minimal adequate model after removal of outlier:

gls([NH_4_] ~ as.factor(Fishing Frequency),

method = "REML")

Coefficient table:

Intercept ± SE (when baseline is for Low fishing frequency): 22.27 ± 8.92, t = 2.50, p = 0.0297.

|  | Low | Medium | High |
| --- | --- | --- | --- |
| Low | - | -4.42 ± 13.38  -0.33  (0.748) | 28.24 ± 12.62  2.24  (0.047) |
| Medium | 4.42 ± 13.38  0.33  (0.748) | - | 32.66 ± 13.38  2.44  (0.033) |
| High | -28.24 ± 12.62  -2.24  (0.047) | -32.66 ± 13.38  -2.44  (0.033) | - |

**Supplementary Model S19 | Nitrite ([NO_2_-N], μM)**

Initial linear regression model:

lm([NO_2_] ~ as.factor(Fishing Frequency))

No minimal adequate model, intercept only (Fishing Frequency, L-ratio = 1.07, d.f. = 4, p = 0.5857)

No minimal adequate model after removal of outlier, intercept only (Fishing Frequency, L-ratio = 0.59, d.f. = 2, p = 0.7435)

**Supplementary Model S20 | Nitrate ([NO_3_-N], μM)**

Initial linear regression model:

lm([NO_3_] ~ as.factor(Fishing Frequency))

No minimal adequate model, intercept only (Fishing Frequency, L-ratio = 1.71, d.f. = 4, p = 0.4249)

No minimal adequate model after removal of outlier, intercept only (Fishing Frequency, L-ratio = 1.69, d.f. = 2, p = 0.4287)

**Supplementary Model S21 | Phosphate ([PO_4_-P], μM)**

Initial linear regression model:

lm([PO_4_] ~ as.factor(Fishing Frequency))

No minimal adequate model, intercept only (Fishing Frequency, L-ratio = 4.77, d.f. = 2, p = 0.0923)

**Supplementary Model S22 | Silicate ([SiO_4_-Si], μM)**

Initial linear regression model:

lm([SiO_4_] ~ as.factor(Fishing Frequency))

No minimal adequate model, intercept only (Fishing Frequency, L-ratio = 4.85, d.f. = 2, p = 0.0884)

ENDS.
