# Supplementary material for: Mediation of macronutrients and carbon by post-disturbance shelf sea sediment communities
Source: Biogeochemistry. 2017 Jun 12;135(1):121–33. doi: 10.1007/s10533-017-0350-9 (PMC6961522; doi:10.1007/s10533-017-0350-9)
Supplement: Supplementary file 2 — Supplementary material 2 (DOCX 99 kb) [file 10533_2017_350_MOESM2_ESM.docx]

**Supplementary Material Part 2 of 2**

**Title: Mediation of macronutrients and carbon by post-disturbance shelf sea sediment communities**

**Journal: Biogeochemistry**

**Authors: Rachel Hale, Jasmin A. Godbold, Jessica Dwight, Christina Wood, Marija Sciberras, Jan G. Hiddink, Martin Solan**

Affiliations:

Rachel Hale, Jasmin A. Godbold, Jessica Dwight, Christina Wood, Martin Solan

Ocean and Earth Science,

National Oceanography Centre Southampton,

University of Southampton Waterfront Campus,

European Way, Southampton, SO14 3ZH

Jasmin A. Godbold

Biological Sciences

Faculty of Natural & Environmental Sciences

Life Sciences Building 85

University of Southampton

Highfield Campus

Southampton, SO17 1BJ

Marija Sciberras, Jan Hiddink

School of Ocean Sciences,

Bangor University,

Menai Bridge, LL59 5AB

Correspondence address:

r.hale@soton.ac.uk

School of Ocean and Earth Science,

National Oceanography Centre Southampton,

University of Southampton Waterfront Campus,

European Way, Southampton, SO14 3ZH

Supplementary Table S3 | The similarity percentage (SIMPER) dissimilarity tables (up to 90% of cumulative differences) of taxa biomass values for cohesive sites showing two level comparisons of similarity within the treatment Fishing Frequency (Levels: low, medium, and high fishing frequency).

Groups tested: high vs medium frequency fishing disturbance. Average site dissimilarity = 80.60

|  | Site: High | Site: Medium |  |  |  |  |
| --- | --- | --- | --- | --- | --- | --- |
| Taxa | Average | Average | Average | Dissimilarity | Percentage | Cumulative |
|  | abundance | abundance | dissimilarity | SD | contribution | percentage |
| *Goneplax rhomboides* | 0 | 0.19 | 24.7 | 0.73 | 30.64 | 30.64 |
| *Nephtys incisa* | 0.04 | 0.03 | 18.69 | 0.92 | 23.19 | 53.83 |
| *Nucula nitidosa* | 0.06 | 0 | 11.97 | 0.46 | 14.85 | 68.69 |
| *Jaxea nocturna* | 0.02 | 0 | 10.31 | 0.62 | 12.79 | 81.47 |
| *Hyala vitrea* | 0.01 | 0.01 | 5.45 | 1.17 | 6.76 | 88.23 |
| *Lagis koreni* | 0 | 0 | 2.66 | 0.54 | 3.3 | 91.53 |

Groups tested: high vs low frequency fishing disturbance. Average site dissimilarity = 57.68

|  | Site: High | Site: Low |  |  |  |  |
| --- | --- | --- | --- | --- | --- | --- |
| Taxa | Average | Average | Average | Dissimilarity | Percentage | Cumulative |
|  | abundance | abundance | dissimilarity | SD | contribution | percentage |
| *Nephtys incisa* | 0.04 | 0.08 | 18.48 | 1.15 | 32.04 | 32.04 |
| *Nucula nitidosa* | 0.06 | 0 | 12.56 | 0.5 | 21.78 | 53.82 |
| *Jaxea nocturna* | 0.02 | 0 | 9.03 | 0.72 | 15.66 | 69.48 |
| *Hyala vitrea* | 0.01 | 0.01 | 5.3 | 1.14 | 9.19 | 78.67 |
| *Notomastus latericeus* | 0 | 0.01 | 4.68 | 0.47 | 8.11 | 86.79 |
| *Lagis koreni* | 0 | 0 | 1.99 | 0.54 | 3.45 | 90.23 |

Groups tested: medium vs low frequency fishing disturbance. Average site dissimilarity = 77.05

|  | Site: Medium | Site: Low |  |  |  |  |
| --- | --- | --- | --- | --- | --- | --- |
| Taxa | Average | Average | Average | Dissimilarity | Percentage | Cumulative |
|  | abundance | abundance | dissimilarity | SD | contribution | percentage |
| *Nephtys incisa* | 0.03 | 0.08 | 35.07 | 1.29 | 45.52 | 45.52 |
| *Goneplax rhomboides* | 0.19 | 0 | 25.41 | 0.75 | 32.97 | 78.5 |
| *Notomastus latericeus* | 0 | 0.01 | 5.07 | 0.44 | 6.58 | 85.08 |
| *Hyala vitrea* | 0.01 | 0.01 | 4.25 | 0.9 | 5.51 | 90.59 |

Supplementary Table S4 | The similarity percentage (SIMPER) dissimilarity tables (up to 90% of cumulative differences) of taxa abundance values for non- cohesive sites showing two level comparisons of similarity within the treatment Fishing Frequency (Levels: low, medium, and high fishing frequency).

Groups tested: low vs medium frequency fishing disturbance. Average site dissimilarity = 77.29

|  | Site: Low | Site: Medium |  |  |  |  |
| --- | --- | --- | --- | --- | --- | --- |
| Taxa | Average | Average | Average | Dissimilarity | Percentage | Cumulative |
|  | abundance | abundance | dissimilarity | SD | contribution | percentage |
| Nematoda | 0.28 | 2.44 | 10.42 | 1.83 | 13.49 | 13.49 |
| *Lagis koreni* | 1.83 | 1.95 | 6.96 | 1.53 | 9 | 22.49 |
| *Poecilochaetus serpens* | 1.27 | 0.65 | 5.54 | 1.45 | 7.17 | 29.66 |
| *Sthenelais limicola* | 1.1 | 0.14 | 4.66 | 2.06 | 6.03 | 35.69 |
| *Magelona minuta* | 0.68 | 0 | 3.33 | 0.99 | 4.3 | 39.99 |
| *Scoloplos armiger* | 0.55 | 0 | 2.32 | 0.78 | 3.01 | 43 |
| *Spio filicornis* | 0.42 | 0 | 2.1 | 1.07 | 2.71 | 45.71 |
| *Magelona mirabilis* | 0.42 | 0.14 | 1.96 | 1 | 2.54 | 48.25 |
| *Microspio mecznikowianus* | 0.36 | 0 | 1.83 | 0.68 | 2.36 | 50.61 |
| Nemertea | 0.14 | 0.36 | 1.72 | 0.87 | 2.22 | 52.84 |
| *Spiophanes bombyx* | 0.28 | 0.28 | 1.71 | 0.86 | 2.21 | 55.05 |
| *Glycera lapidum* | 0 | 0.28 | 1.61 | 0.76 | 2.08 | 57.13 |
| *Echinocyamus pusillus* | 0 | 0.28 | 1.51 | 0.77 | 1.95 | 59.08 |
| *Malmgrenia arenicolae* | 0.28 | 0 | 1.48 | 0.77 | 1.91 | 60.99 |
| *Syllis cornuta* | 0 | 0.32 | 1.32 | 0.48 | 1.71 | 62.7 |
| *Spisula elliptica* | 0 | 0.22 | 1.26 | 0.47 | 1.64 | 64.33 |
| *Grania* | 0 | 0.28 | 1.22 | 0.48 | 1.58 | 65.91 |
| *Nephtys kersavelensis* | 0.22 | 0 | 1.04 | 0.48 | 1.35 | 67.26 |
| *Arctica islandica* | 0.14 | 0.14 | 1.04 | 0.66 | 1.34 | 68.6 |
| *Phoronis* | 0.14 | 0.14 | 1.03 | 0.66 | 1.33 | 69.93 |
| *Mediomastus fragilis* | 0.14 | 0.14 | 1 | 0.66 | 1.3 | 71.23 |
| *Streptosyllis bidentata* | 0 | 0.22 | 0.97 | 0.48 | 1.25 | 72.48 |
| *Magelona wilsoni* | 0.14 | 0 | 0.94 | 0.48 | 1.22 | 73.7 |
| *Hesionura elongata* | 0.14 | 0.14 | 0.93 | 0.66 | 1.21 | 74.91 |
| *Euspira nitida* | 0 | 0.14 | 0.9 | 0.47 | 1.17 | 76.08 |
| *Musculus discors* juv. | 0 | 0.14 | 0.9 | 0.47 | 1.17 | 77.24 |
| *Pseudomystides limbata* | 0 | 0.22 | 0.9 | 0.48 | 1.16 | 78.41 |
| *Dosinia exoleta* | 0.14 | 0 | 0.83 | 0.48 | 1.08 | 79.49 |
| Spatangoidea juv. | 0.22 | 0 | 0.8 | 0.49 | 1.04 | 80.52 |
| *Abra prismatica* | 0 | 0.14 | 0.8 | 0.47 | 1.03 | 81.56 |
| *Mya arenaria* juv. | 0 | 0.14 | 0.71 | 0.48 | 0.92 | 82.47 |
| *Monopseudocuma gilsoni* | 0 | 0.14 | 0.71 | 0.48 | 0.92 | 83.39 |
| Tanaiadacea | 0 | 0.14 | 0.71 | 0.48 | 0.92 | 84.3 |
| Asteroidea juv. | 0 | 0.14 | 0.71 | 0.48 | 0.92 | 85.22 |
| *Amphictene auricoma* | 0.14 | 0 | 0.66 | 0.48 | 0.85 | 86.07 |
| *Chaetozone setosa* | 0.14 | 0 | 0.66 | 0.48 | 0.85 | 86.92 |
| Nereidiidae juv. | 0.14 | 0 | 0.66 | 0.48 | 0.85 | 87.76 |
| Paraonidae indet. | 0.14 | 0 | 0.66 | 0.48 | 0.85 | 88.61 |
| *Kurtiella bidentata* | 0.14 | 0 | 0.66 | 0.48 | 0.85 | 89.46 |
| *Bodotria arenosa* | 0.14 | 0 | 0.66 | 0.48 | 0.85 | 90.31 |

Groups tested: low vs high frequency fishing disturbance. Average site dissimilarity = 82.31

|  | Site: Low | Site: High |  |  |  |  |
| --- | --- | --- | --- | --- | --- | --- |
| Taxa | Average | Average | Average | Dissimilarity | Percentage | Cumulative |
|  | abundance | abundance | dissimilarity | SD | contribution | percentage |
| *Lagis koreni* | 1.83 | 0.36 | 9.81 | 1.4 | 11.91 | 11.91 |
| *Poecilochaetus serpens* | 1.27 | 0.28 | 6.11 | 1.48 | 7.42 | 19.33 |
| *Ophelina acuminata* | 0 | 0.74 | 4.93 | 0.93 | 5.99 | 25.33 |
| *Magelona minuta* | 0.68 | 0 | 4.13 | 1 | 5.02 | 30.35 |
| *Abra nitida* | 0 | 0.64 | 4.05 | 1.06 | 4.92 | 35.26 |
| *Sthenelais limicola* | 1.1 | 0.72 | 3.22 | 1.04 | 3.92 | 39.18 |
| *Scoloplos armiger* | 0.55 | 0.14 | 3.16 | 0.95 | 3.84 | 43.02 |
| *Magelona mirabilis* | 0.42 | 0 | 2.65 | 1.05 | 3.21 | 46.24 |
| *Spio filicornis* | 0.42 | 0 | 2.65 | 1.05 | 3.21 | 49.45 |
| Nematoda | 0.28 | 0.42 | 2.31 | 0.98 | 2.81 | 52.26 |
| *Microspio mecznikowianus* | 0.36 | 0 | 2.3 | 0.67 | 2.8 | 55.05 |
| Spatangoidea juv. | 0.22 | 0.22 | 1.91 | 0.64 | 2.33 | 57.38 |
| *Tellimya ferruginosa* | 0 | 0.28 | 1.91 | 0.47 | 2.32 | 59.7 |
| *Malmgrenia arenicolae* | 0.28 | 0 | 1.87 | 0.78 | 2.27 | 61.97 |
| Nemertea | 0.14 | 0.14 | 1.51 | 0.62 | 1.83 | 63.8 |
| *Dosinia exoleta* | 0.14 | 0.14 | 1.49 | 0.64 | 1.81 | 65.62 |
| *Spiophanes bombyx* | 0.28 | 0 | 1.39 | 0.78 | 1.69 | 67.31 |
| *Exogone hebes* | 0 | 0.22 | 1.38 | 0.47 | 1.67 | 68.98 |
| *Chaetozone setosa* | 0.14 | 0.14 | 1.34 | 0.65 | 1.63 | 70.61 |
| *Nephtys kersavelensis* | 0.22 | 0 | 1.27 | 0.49 | 1.55 | 72.16 |
| *Magelona wilsoni* | 0.14 | 0 | 1.27 | 0.48 | 1.54 | 73.7 |
| Edwardsiidae | 0 | 0.14 | 1.08 | 0.46 | 1.32 | 75.01 |
| *Cylichna cylindracea* | 0 | 0.14 | 0.96 | 0.47 | 1.16 | 76.18 |
| *Echinocardium cordatum* | 0 | 0.14 | 0.96 | 0.47 | 1.16 | 77.34 |
| *Nephtys hombergii* | 0 | 0.14 | 0.87 | 0.47 | 1.05 | 78.39 |
| *Nephtys hystricis* | 0 | 0.14 | 0.87 | 0.47 | 1.05 | 79.45 |
| *Upogebia pusilla* | 0 | 0.14 | 0.87 | 0.47 | 1.05 | 80.5 |
| *Eumida* sp. | 0 | 0.14 | 0.84 | 0.47 | 1.03 | 81.53 |
| *Nephtys* sp. | 0 | 0.14 | 0.84 | 0.47 | 1.03 | 82.55 |
| *Gari fervensis* | 0 | 0.14 | 0.84 | 0.47 | 1.03 | 83.58 |
| *Pseudocuma longicornis* | 0 | 0.14 | 0.84 | 0.47 | 1.03 | 84.61 |
| *Amphictene auricoma* | 0.14 | 0 | 0.8 | 0.49 | 0.98 | 85.58 |
| Nereidiidae juv. | 0.14 | 0 | 0.8 | 0.49 | 0.98 | 86.56 |
| Paraonidae indet. | 0.14 | 0 | 0.8 | 0.49 | 0.98 | 87.53 |
| *Arctica islandica* | 0.14 | 0 | 0.8 | 0.49 | 0.98 | 88.51 |
| *Kurtiella bidentata* | 0.14 | 0 | 0.8 | 0.49 | 0.98 | 89.48 |
| *Bodotria arenosa* | 0.14 | 0 | 0.8 | 0.49 | 0.98 | 90.46 |

Groups tested: medium vs high frequency fishing disturbance. Average site dissimilarity = 87.27

|  | Site: Medium | Site: High |  |  |  |  |
| --- | --- | --- | --- | --- | --- | --- |
| Taxa | Average | Average | Average | Dissimilarity | Percentage | Cumulative |
|  | abundance | abundance | dissimilarity | SD | contribution | percentage |
| *Lagis koreni* | 1.95 | 0.36 | 11.67 | 1.53 | 13.37 | 13.37 |
| Nematoda | 2.44 | 0.42 | 11.52 | 1.85 | 13.2 | 26.58 |
| *Ophelina acuminata* | 0 | 0.74 | 4.77 | 0.97 | 5.47 | 32.05 |
| *Abra nitida* | 0 | 0.64 | 3.93 | 1.09 | 4.5 | 36.55 |
| *Poecilochaetus serpens* | 0.65 | 0.28 | 3.86 | 0.89 | 4.43 | 40.97 |
| *Sthenelais limicola* | 0.14 | 0.72 | 3.86 | 1.39 | 4.42 | 45.39 |
| Nemertea | 0.36 | 0.14 | 2.19 | 0.87 | 2.5 | 47.9 |
| *Glycera lapidum* | 0.28 | 0 | 2.01 | 0.78 | 2.3 | 50.2 |
| *Grania* | 0.28 | 0.14 | 1.95 | 0.67 | 2.23 | 52.43 |
| *Spiophanes bombyx* | 0.28 | 0 | 1.87 | 0.76 | 2.14 | 54.57 |
| *Echinocyamus pusillus* | 0.28 | 0 | 1.85 | 0.79 | 2.12 | 56.69 |
| *Tellimya ferruginosa* | 0 | 0.28 | 1.85 | 0.48 | 2.12 | 58.81 |
| *Exogone hebes* | 0.14 | 0.22 | 1.78 | 0.65 | 2.04 | 60.85 |
| *Spisula elliptica* | 0.22 | 0 | 1.57 | 0.49 | 1.8 | 62.65 |
| *Syllis cornuta* | 0.32 | 0 | 1.55 | 0.49 | 1.77 | 64.42 |
| *Spatangoidea juv.* | 0 | 0.22 | 1.3 | 0.48 | 1.49 | 65.92 |
| *Nephtys* sp. | 0.14 | 0.14 | 1.23 | 0.66 | 1.41 | 67.33 |
| *Thracia villosiuscula* | 0.14 | 0.14 | 1.22 | 0.66 | 1.4 | 68.73 |
| *Euspira nitida* | 0.14 | 0 | 1.15 | 0.48 | 1.32 | 70.05 |
| *Musculus discors* juv. | 0.14 | 0 | 1.15 | 0.48 | 1.32 | 71.37 |
| *Streptosyllis bidentata* | 0.22 | 0 | 1.14 | 0.49 | 1.31 | 72.68 |
| *Pseudomystides limbata* | 0.22 | 0 | 1.06 | 0.49 | 1.21 | 73.89 |
| Edwardsiidae | 0 | 0.14 | 1.04 | 0.47 | 1.19 | 75.08 |
| *Magelona mirabilis* | 0.14 | 0 | 0.99 | 0.49 | 1.14 | 76.21 |
| *Abra prismatica* | 0.14 | 0 | 0.99 | 0.49 | 1.14 | 77.35 |
| *Scoloplos armiger* | 0 | 0.14 | 0.92 | 0.48 | 1.06 | 78.41 |
| *Cylichna cylindracea* | 0 | 0.14 | 0.92 | 0.48 | 1.06 | 79.47 |
| *Echinocardium cordatum* | 0 | 0.14 | 0.92 | 0.48 | 1.06 | 80.53 |
| *Mya arenaria* juv. | 0.14 | 0 | 0.86 | 0.49 | 0.98 | 81.51 |
| *Monopseudocuma gilsoni* | 0.14 | 0 | 0.86 | 0.49 | 0.98 | 82.5 |
| Tanaiadacea | 0.14 | 0 | 0.86 | 0.49 | 0.98 | 83.48 |
| Asteroidea juv. | 0.14 | 0 | 0.86 | 0.49 | 0.98 | 84.47 |
| *Nephtys hombergii* | 0 | 0.14 | 0.84 | 0.48 | 0.97 | 85.43 |
| *Nephtys hystricis* | 0 | 0.14 | 0.84 | 0.48 | 0.97 | 86.4 |
| *Upogebia pusilla* | 0 | 0.14 | 0.84 | 0.48 | 0.97 | 87.37 |
| *Chaetozone setosa* | 0 | 0.14 | 0.82 | 0.48 | 0.94 | 88.31 |
| *Eumida* sp. | 0 | 0.14 | 0.82 | 0.48 | 0.94 | 89.25 |
| *Gari fervensis* | 0 | 0.14 | 0.82 | 0.48 | 0.94 | 90.2 |

Supplementary Table S5 | The similarity percentage (SIMPER) dissimilarity tables (up to 90% of cumulative differences) of taxa biomass values for non-cohesive sites showing two level comparisons of similarity within the treatment Fishing Frequency (Levels: low, medium, and high fishing frequency).

Groups tested: low vs medium frequency fishing disturbance. Average site dissimilarity = 85.79

|  | Site: Low | Site: Medium |  |  |  |  |
| --- | --- | --- | --- | --- | --- | --- |
| Taxa | Average | Average | Average | Dissimilarity | Percentage | Cumulative |
|  | abundance | abundance | dissimilarity | SD | contribution | percentage |
| *Lagis koreni* | 0.03 | 0.05 | 23.02 | 1.1 | 26.83 | 26.83 |
| *Ensis ensis* | 0 | 0.66 | 12 | 0.49 | 13.99 | 40.82 |
| *Euspira nitida* | 0 | 0.01 | 8.17 | 0.45 | 9.52 | 50.34 |
| *Sthenelais limicola* | 0.01 | 0.01 | 7.86 | 0.89 | 9.17 | 59.51 |
| *Glycymeris glycymeris* | 0 | 0.41 | 7.53 | 0.49 | 8.77 | 68.28 |
| Nemertea | 0 | 0.01 | 4.19 | 0.46 | 4.88 | 73.16 |
| *Dosinia exoleta* | 0.01 | 0 | 3.48 | 0.42 | 4.06 | 77.22 |
| *Microspio mecznikowianus* | 0.01 | 0 | 3.28 | 0.43 | 3.82 | 81.04 |
| *Scoloplos armiger* | 0.01 | 0 | 2.13 | 0.54 | 2.49 | 83.53 |
| *Corystes cassivelaunus* | 0.01 | 0 | 2.11 | 0.42 | 2.46 | 85.99 |
| *Nephtys kersavelensis* | 0.01 | 0 | 1.98 | 0.42 | 2.31 | 88.3 |
| *Magelona mirabilis* | 0 | 0 | 1.94 | 0.88 | 2.26 | 90.56 |

Groups tested: low vs high frequency fishing disturbance. Average site dissimilarity = 96.26

|  | Site: Low | Site: High |  |  |  |  |
| --- | --- | --- | --- | --- | --- | --- |
| Taxa | Average | Average | Average | Dissimilarity | Percentage | Cumulative |
|  | abundance | abundance | dissimilarity | SD | contribution | percentage |
| *Echinocardium cordatum* | 0 | 0.53 | 19.27 | 0.49 | 20.02 | 20.02 |
| *Gari fervensis* | 0 | 0.29 | 18.62 | 0.49 | 19.35 | 39.37 |
| Edwardsiidae | 0 | 0.06 | 13.92 | 0.48 | 14.46 | 53.83 |
| *Nephtys hombergii* | 0 | 0.1 | 9.49 | 0.49 | 9.86 | 63.69 |
| *Upogebia pusilla* | 0 | 0.09 | 8.47 | 0.49 | 8.8 | 72.49 |
| *Dosinia exoleta* | 0.01 | 0.01 | 7.69 | 0.47 | 7.99 | 80.48 |
| *Lagis koreni* | 0.03 | 0 | 4.44 | 0.56 | 4.61 | 85.09 |
| *Microspio mecznikowianus* | 0.01 | 0 | 1.74 | 0.37 | 1.8 | 86.89 |
| *Sthenelais limicola* | 0.01 | 0 | 1.61 | 0.6 | 1.68 | 88.57 |
| *Ophelina acuminata* | 0 | 0 | 1.22 | 0.8 | 1.27 | 89.84 |
| Nemertea | 0 | 0 | 1.18 | 0.48 | 1.23 | 91.07 |

Groups tested: medium vs high frequency fishing disturbance. Average site dissimilarity = 98.42

|  | Site: Medium | Site: High |  |  |  |  |
| --- | --- | --- | --- | --- | --- | --- |
| Taxa | Average | Average | Average | Dissimilarity | Percentage | Cumulative |
|  | abundance | abundance | dissimilarity | SD | contribution | percentage |
| *Echinocardium cordatum* | 0 | 0.53 | 16.6 | 0.46 | 16.86 | 16.86 |
| *Gari fervensis* | 0 | 0.29 | 15.48 | 0.45 | 15.73 | 32.59 |
| *Edwardsiidae* | 0 | 0.06 | 10.58 | 0.43 | 10.75 | 43.34 |
| *Ensis ensis* | 0.66 | 0 | 10.33 | 0.48 | 10.5 | 53.84 |
| *Lagis koreni* | 0.05 | 0 | 7.99 | 0.84 | 8.12 | 61.96 |
| *Nephtys hombergii* | 0 | 0.1 | 7.7 | 0.45 | 7.82 | 69.78 |
| *Upogebia pusilla* | 0 | 0.09 | 6.87 | 0.45 | 6.98 | 76.77 |
| *Glycymeris glycymeris* | 0.41 | 0 | 6.48 | 0.48 | 6.59 | 83.35 |
| *Dosinia exoleta* | 0 | 0.01 | 5.07 | 0.42 | 5.15 | 88.51 |
| *Euspira nitida* | 0.01 | 0 | 2.55 | 0.34 | 2.59 | 91.1 |
